# Chloroplast-encoded serotonin *N*-acetyltransferase in the red alga *Pyropia yezoensis*: gene transition to the nucleus from chloroplasts

**DOI:** 10.1093/jxb/eru357

**Published:** 2014-09-02

**Authors:** Yeong Byeon, Hyoung Yool Lee, Dong-Woog Choi, Kyoungwhan Back

**Affiliations:** ^1^Department of Biotechnology, Interdisciplinary Program of Bioenergy and Biomaterials, Bioenergy Research Center, College of Agriculture and Life Sciences, Chonnam National University, Gwangju, Republic of Korea; ^2^Department of Biology Education, Chonnam National University, Gwangju 500-757, Republic of Korea

**Keywords:** Endosymbiosis, laver, melatonin, *Pyropia*, red algae, serotonin N-acetyltransferase

## Abstract

Melatonin biosynthesis involves the *N*-acetylation of arylalkylamines such as serotonin, which is catalysed by serotonin *N*-acetyltransferase (SNAT), the penultimate enzyme of melatonin biosynthesis in both animals and plants. Here, we report the functional characterization of a putative *N*-acetyltransferase gene in the chloroplast genome of the alga laver (*Pyropia yezoensis*, formerly known as *Porphyra yezoensis*) with homology to the rice *SNAT* gene. To confirm that the putative *Pyropia yezoensis SNAT* (*PySNAT*) gene encodes an SNAT, we cloned the full-length chloroplastidic *PySNAT* gene by PCR and purified the recombinant PySNAT protein from *Escherichia coli*. PySNAT was 174 aa and had 50% amino acid identity with cyanobacteria SNAT. Purified recombinant PySNAT showed a peak activity at 55 °C with a *K*_m_ of 467 µM and *V*_max_ of 28 nmol min–1 mg^–1^ of protein. Unlike other plant SNATs, PySNAT localized to the cytoplasm due to a lack of N-terminal chloroplast transit peptides. Melatonin was present at 0.16ng g^–1^ of fresh mass but increased during heat stress. Phylogenetic analysis of the sequence suggested that *PySNAT* has evolved from the cyanobacteria *SNAT* gene via endosymbiotic gene transfer. Additionally, the chloroplast transit peptides of plant SNATs were acquired 1500 million years ago, concurrent with the appearance of green algae.

## Summary

The *SNAT* gene of the red alga *Pyropia yezoensis* is chloroplast encoded, suggesting that plant *SNAT* genes evolved directly from cyanobacteria to red algae via endosymbiosis and thereafter were transferred to the nucleus.

## Introduction

*Pyropia yezoensis*, a marine alga laver known previously as *Porphyra yezoensis* (Sutherland *et al.*, 2011), is used commercially in foods such as sushi. The products of laver are valued at around US$2 billion within East Asia (Sahoo *et al.*, 2002). Due to its economic importance and nutritional properties, *Pyropia* cultivation is expanding to other countries (Noda, 1993). Around 75 species of *Pyropia* have been reported globally, and they can grow up to several metres in length with a linear or orbicular morphology. The thalli are in the gametophytic phase and consist of one or two thick cells containing one or two chloroplasts (Sahoo *et al.*, 2002; Sutherland *et al.*, 2011). In particular, *Pyropia* is known to be higher in vitamin C than oranges (Noda, 1993).

Melatonin was first isolated from beef pineal glands as an active ingredient for preventing the darkening of frog skin (Lerner *et al.*, 1958). Since then, melatonin has been found to play pleiotropic roles in animals, including circadian rhythmicity (Reiter, 1991), seasonal reproduction (Barrett and Bolborea, 2012), the scavenging of free radicals (García *et al.*, 2014), enhancing innate immune responses (Calvo *et al.*, 2013), and delaying the aging process (Hardeland, 2013). In contrast to animals, the presence of melatonin in plants was first uncovered in 1995 from various tissues and fruits (Dubbels *et al.*, 1995; Hattori *et al.*, 1995). Several reports over the past two decades have implicated plant melatonin in numerous biological activities, including root and leaf growth (Hernández-Ruiz *et al.*, 2005; Chen *et al.*, 2009; Park and Back, 2012; Pelagio-Flores *et al.*, 2012; Sarropoulou *et al.*, 2012), the promotion of germination (Tiryaki and Keles, 2012; Zhang *et al.*, 2013), the delay of flowering (Kolář *et al.*, 2003; Byeon and Back, 2014*a*), and increasing photosynthesis (Lazár *et al.*, 2013). In addition, melatonin is generally thought to be involved in plant defence responses to various stresses such as cold (Posmyk *et al.*, 2009; Kang *et al.*, 2010; Bajwa *et al.*, 2014), drought (Wang *et al.*, 2013*b*; Zhang *et al.*, 2013), herbicides (Park *et al.*, 2013c), salt (Li *et al.*, 2012), heavy metals (Tan *et al.*, 2007*a*), senescence (Wang *et al*., 2012, 2013 *a*), and pathogens (Yin *et al.*, 2013). However, the precise role of melatonin in plant growth and development remains unclear. The role of melatonin has been explored using molecular genetic analysis of a melatonin-deficient mutant line and gain-of-function analysis with melatonin-rich transgenic plants (Kang *et al*., 2010; Okazaki *et al.*, 2010; Byeon and Back, 2014*a*; Wang *et al.*, 2014), which revealed similar functions to those mentioned above (Paredes *et al.*, 2009).

In accordance with the pleiotropic roles of melatonin in plants, melatonin levels vary significantly among plant species, ranging from a few picograms to micrograms per gram of mass (Chen *et al.*, 2003; Rodriguez-Naranjo *et al.*, 2011; Ramakrishna *et al.*, 2012). On the other hand, melatonin has been found in all plant lineages including cyanobacteria, which are thought to be the origin of chloroplasts (Tilden *et al.*, 1997; Tan *et al.*, 2013), Charophyceae (*Chara australis*), Chlorophyceae (*Chlamydomonas* spp., *Dunaliella tertiolecta*, and *Acetabularia acetabulum*), Phaeophyceae (*Pterygophora califirnia*, *Laminaria digitata*, and *Petalonia fascia*), Rhodophyceae (*Gracilaria tenustipitata*, *Palmaria palmate*, and *Pyropia umbilicalis*), alveolate (e.g. *Gonyaulax polyedra*, *Alexandrium lustitanicum*, *Ceratium horridum*, *and Pyrocyctis lunula*), and excavate (*Euglena gracilis*) (Balzer and Hardeland, 1991; Fuhrberg *et al.*, 1996; Lorenz and Lüning, 1998; Hardeland, 1999; Tal *et al.*, 2011). Like melatonin, which exists in all plant kingdom as described above, serotonin *N*-acetyltransferase (SNAT), the penultimate enzyme for melatonin biosynthesis in plants, is conserved throughout evolutionary plant lineages (Byeon *et al.*, 2013; Kang *et al.*, 2013). Homologues of *SNAT* genes are found in cyanobacteria, Chlorophyceae (*Chlamydomonas* reinhardtii and *Volvox carteri*), Rhodophyceae (*Pyropia yezoensis* and *Cyanidioschyzon merolae* strain 10D), Prasinophyceae (*Ostreococcus tauri*), bryophytes (*Physcomitrella patens*), Lycopodiophyta (*Selaginella moellendorffii*), and Euphyllophyta (e.g. *Picea sitchensis*, *Arabidopsis thaliana*, and *Oryza sativa*). Among these *SNAT* homologues, two *SNAT* genes from cyanobacteria (Byeon *et al.*, 2013) and rice (Kang *et al.*, 2013) were functionally characterized. Unexpectedly, a *SNAT* homologue from the alga laver was found in the plastid genome, whereas other *SNAT* genes from green algae to higher plants are present in the host nuclear genomes. This suggests that the plant *SNAT* gene originated from the incorporation of cyanobacteria into red algae via an endosymbiosis process.

In this study, we investigated whether the chloroplast-encoded *SNAT* homologue of the alga laver has SNAT activity and whether its activity is associated with melatonin production.

## Materials and methods

### Laver material and growth conditions

Laver (*Pyropia yezoensis*) was cultivated as follows. Leafy gametophytes of *Pyropia yezoensis* were cultured in modified Grund medium (McLachlan, 1973) at 10 °C with a photon flux density of 80 µmol photon m^–2^ s^–1^ provided by cool-white fluorescent lamps with a photoperiod of 14/10 (light/dark) in a growth chamber. For high-temperature treatment, cultivation bottles growing *Pyropia yezoensis* were transferred to a 25 °C growth chamber under the same light conditions described above.

### Isolation of *Pyropia yezoensis* genomic DNA and *PySNAT* cloning

For genomic DNA extraction, leafy gametophytes of *Pyropia yezoensis* (100mg) were ground to a powder in liquid nitrogen using a mortar and pestle, and DNA was extracted with the buffer provided by a DNeasy Plant Mini kit (Qiagen, Tokyo, Japan). The full-length *PySNAT* sequence was cloned by PCR using the genomic DNA of *Pyropia yezoensis* with a primer set based on *PySNAT* sequence information (GenBank accession no. NC_007932). The forward and reverse primers were 5′-ATGATCTTCTGGAAAAAT-3′ and 5′-TTATCTAGGATACCAAAA-3′, respectively. The resulting PCR-amplified *PySNAT* product was ligated into the pTOP Blunt V2 vector to generate pTOP blunt V2:*PySNAT*, and the sequence of *PtSNAT* was verified.

### Construction of *Escherichia coli* expression vectors for PySNAT protein purification

The pET300 Gateway® vector (Invitrogen, Carlsbad, CA, USA) was used for N-terminal His-tagged expression of PySNAT. Full-length *PySNAT* and truncated ∆22*PySNAT* (lacking the N-terminal 22 aa) were amplified by PCR using appropriate primers harbouring the *attB* recombination sequences. For the full-length *PySNAT*, the primer set was as follows: forward primer 5′-AAAAAGCAGGCTCCATGATCTTCTGGAAAA-3′; reverse primer 5′-AGAAAGCTGGGTTTATCTAGGATACCAAAA-3′. The forward primer for the truncated ∆22*PySNAT* was 5′-AAAAAGCAGGCTCCATGAAACTTATTGTTTTAG-3′ and the reverse primer was the same as above. The resulting products were cloned into the pDONR221 Gateway vector (Invitrogen). The pDONR221-PySNAT and pDONR221-∆22PySNAT entry vectors were then recombined with the pET300 Gateway destination vector via LR recombination to generate pET300-PySNAT and pET300-∆22PySNAT. The pET28b vector (Novagen, Madison, WI, USA) was used for C-terminal His-tagged expression of *PySNAT*. The full-length *PySNAT* gene was amplified by PCR using 5′-AAAAGCAGGCCC **ATG** GTCTTCTGGAAAAAT-3′ as the forward primer (*Nco*I restriction sites are underlined and the translation start codon is in bold), 5′-GTGCTCGAGTCTAGGATACCAAAA-3′ (the *Xho*I site is underlined) as the reverse primer, and pTOP blunt V2:*PySNAT* as the template. Truncated *PySNAT* (∆22*PySNAT*) was amplified by PCR using 5′-ACC **ATG** GCAAAACTTATTGTTTTA-3′ as the forward primer (*Nco*I restriction sites are underlined and the translation start codon is in bold) and 5′-GTGCTCGAGTCTAGGATACCAAAA-3′ (the *Xho*I site is underlined) as the reverse primer. The PCR products were cloned into a T&A vector (RBC Bioscience, New Taipei City, Taiwan), digested with *Nco*I and *Xho*I, gel purified, and ligated into the same restriction sites in the pET28(b) reading frame. *E. coli* BL21(DE3) was used as the host strain for both pET300 and pET28(b) containing the *PySNAT* genes. Cell culture and purification procedures using a Ni-NTA column were performed according to the manufacturer’s instructions (Qiagen). The purified recombinant PySNAT proteins were dissolved in 10 mM Tris/HCl (pH 8.0) and 50% glycerol solution and stored at –20°C until further analysis.

### Analyses for SNAT enzyme activity and enzyme kinetics

Purified recombinant PySNAT proteins were incubated in a total volume of 100 µl containing 0.5mM serotonin and 0.5mM acetyl-CoA in 100mM potassium phosphate (pH 8.8), as described previously (Byeon *et al.*, 2013). Briefly, all SNAT assays were conducted at 55 °C (or varying temperatures) for 1h and stopped by the addition of 50 µl of methanol. A 10 µl aliquot was subjected to high-performance liquid chromatography (HPLC) using the fluorescence detector system, as described previously (Byeon *et al.*, 2014). Non-enzymatic reaction products, which were generated in the absence of the PySNAT enzyme, were subtracted. The substrate affinity (*K*_m_) and maximum reaction rate (*V*_max_) were calculated from Lineweaver–Burk plots. The protein concentration was determined using the Bradford method and a protein assay dye (Bio-Rad, Hercules, CA, USA). The analysis was performed in triplicate.

### Subcellular localization of PySNAT

Confocal microscopic analysis was performed to investigate the subcellular localization of PySNAT. The pER-mCherry vector was used for subcellular localization analysis (a kind gift from Dr H. G. Kang, Texas State University, San Marcos, TX, USA). Full-length *PySNAT* cDNA was amplified by PCR with the primer set harbouring an *Asc*I site (forward 5′-GGGGGCGCGCCATGATCTTCTGGAAAA-3′; reverse 5′-GGGGGCGCGCCTCTAGGATACCAAAA-3′). The resulting PCR product was cloned into the TA vector (RBC Bioscience) and the *PtSNAT* insert was digested using the *Asc*I restriction endonuclease, purified, and ligated into the *Asc*I site of the binary vector pER8-mCherry containing the oestrogen-inducible XVE promoter (Pxve) to create pER8-PySNAT-mCherry. The pER-PySNAT-mCherry plasmid was transformed into *Agrobacterium tumefaciens* strain GV2260 using the freeze–thaw method and transient expression analyses were performed as described by Voinnet *et al.* (2003). The pBIN61-GFP plasmid was used as a cytosolic expression marker (Byeon *et al.*, 2014). *Nicotiana benthamiana* leaves were infiltrated with *Agrobacterium* strains and then induced with β-estradiol (10 µm) by infiltration as described previously (Byeon *et al.*, 2014). Images were acquired using a Leica TCS-SP5 confocal microscope with the Leica LAS-AF software version 1.8.2. (Leica Microsystems, Exton, PA, USA). Green fluorescent protein (GFP) was excited with a blue argon ion laser (488nm), and emitted light was collected between 494 and 546nm. mCherry was excited with an orange He-Ne laser (594nm), and emitted light was collected from 576 to 629nm. Chloroplasts were excited with a blue argon laser (488nm), and emitted light was collected from 660 to 731nm. Signals were collected separately and later superimposed.

### HPLC analysis of melatonin

The frozen *Pyropia yezoensis* tissue samples (100mg) were ground to a powder in liquid nitrogen using TissueLyser II (Qiagen) and extracted with 1.5ml of chloroform overnight at 4 °C. The chloroform extracts were evaporated and dissolved in 1.5ml of 35% methanol. Aliquots of 10 µl were subjected to HPLC with a fluorescence detector system (Waters, Milford, MA, USA). The samples were separated on a Sunfire C18 column (4.6×150mm; Waters) with a gradient elution profile (from 42 to 50% methanol in 0.1% formic acid for 27min, followed by isocratic elution with 50% methanol in 0.1% formic acid for 18min at a flow rate of 0.15ml min^–1^). Melatonin was detected at 280nm excitation and 348nm emission.

### Prediction of chloroplast transit peptides

The network-based method (ChloroP) was used to identify chloroplast transit peptides and their cleavage sites from various N-terminal sequences of SNAT homologues (Emanuelsson *et al.*, 1999). The ChloroP predictor is available as a web server at http://www.cbs.dtu.dk/services/ChloroP/. A phylogenetic tree was generated using BLAST-Explorer (Dereeper *et al.*, 2010).

### Statistical analysis

One-way analysis of variance was used for all statistical evaluations. *P*<0.05 was considered to indicate statistical significance.

## Results

### Characterization of the *SNAT* gene in the *Pyropia yezoensis* chloroplast genome

**Fig. 1.**
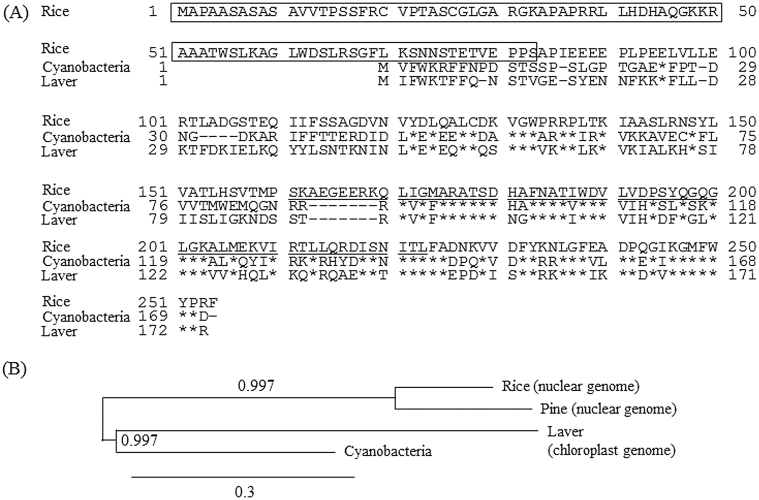
(A) Comparison of the deduced amino acid sequences of SNAT from rice (GenBank accession no. AK059369), cyanobacteria (NP_442603), and laver (NC_007932). The conserved acetyl-CoA binding motif is underlined. The asterisks denote identity. Dashes indicate gaps that were introduced to maximize homology. Amino acid residues predicted to be chloroplast transit sequences are boxed. (B) Phylogenetic analysis of SNAT homologues from rice, loblolly pine, cyanobacteria, and laver. Numbers denote branch support values of each node. Bar, 0.3 substitutions per site.

The rice *SNAT* gene exists as a single copy in the rice genome and is highly conserved in plant lineages, including cyanobacteria (Byeon *et al.*, 2013). According to BLAST analysis, plant *SNAT* homologues have been found in the nuclear genome in all members of Chlorophyta that were examined, such as green algae and terrestrial plants, but the *SNAT* homologue was located in chloroplasts in rhodophyta, including red algae such as laver (*Pyropia yezoensis*). Therefore, we explored whether the chloroplast-encoded *SNAT* in red algae showed SNAT enzyme activity. We cloned the chloroplast-encoded *SNAT* homologue from *Pyropia yezoensis* (*PySNAT*). PySNAT was 174 aa with a calculated molecular weight of approximately 20099Da (Fig. 1). The PySNAT polypeptides shared 50% identity with the cyanobacteria SNAT and also showed high identity to rice and loblolly pine at 45 and 43%, respectively, when compared with the level of mature polypeptides of rice and loblolly pine. The theoretical isoelectric point (pI) was higher in PySNAT (9.36) than cyanobacteria SNAT (pI 7.78), but G+C content decreased to 29% in *PySNAT* relative to 51% in cyanobacteria *SNAT*. Phylogenetic analysis revealed that PySNAT is placed in the same clade as the cyanobacteria SNAT, suggesting that PySNAT originated from cyanobacteria via endosymbiosis. Unlike loblolly pine and rice SNAT polypeptides, no N-terminal extension peptides were observed as a chloroplast transit peptide.

### Bacterial expression and purification of PySNAT

**Fig. 2.**
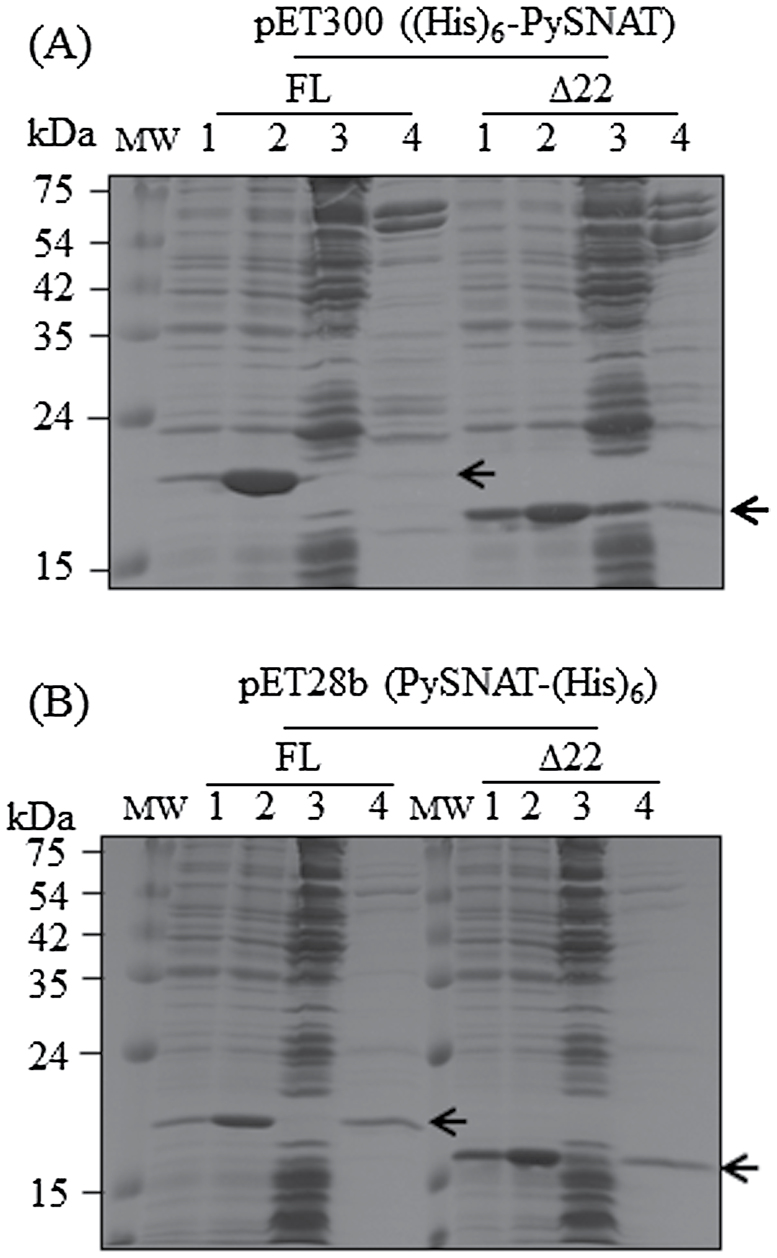
(A) Expression of the N-terminal His-tagged *PySNAT* gene (pET300-PySNAT and pET300-∆22PySNAT) in *E. coli* and its affinity purification. (B) Expression and purification of the C-terminal His-tagged *PySNAT* gene (pET28b-PySNAT and pET28b-∆22PySNAT) in *E. coli*. Each recombinant plasmid was transformed into *E*. *coli* host strain BL21(DE3) expressing full-length (FL) or truncated (∆22) *PySNAT*. The recombinant *E. coli* strains were incubated with or without IPTG before total and soluble protein extraction. Proteins were separated by 12% SDS-PAGE and stained with Coomassie blue. MW, molecular mass standards; lane 1, total protein in 15 µl aliquots of bacterial cells without IPTG; lane 2, total protein after IPTG treatment; lane 3, 20 µg soluble protein; lane 4, PySNAT proteins (3 µg) purified by affinity chromatography (arrows).

**Fig. 3.**
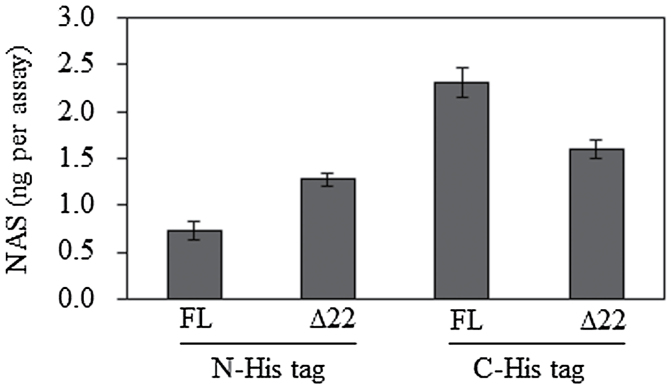
SNAT enzyme activity measurement of various recombinant PySNAT proteins. The SNAT enzyme assay was performed at 30 °C in the presence of 0.5mM serotonin and 0.5mM acetyl-CoA for 30min. The reaction product (NAS) was quantified by HPLC as described in Materials and methods.

An *E. coli* heterologous expression system was used to purify the PySNAT protein. To facilitate the purification of PySNAT, we utilized a His-tagged affinity purification system with either pET300 or pET28b vectors. pET300 generated an N-terminal His-tagged PySNAT, whereas pET28b resulted in a C-terminal His-tagged PySNAT (Fig. 2). Full-length PySNAT expression using the N-terminal His-tagged system yielded high expression after isopropyl β-d-thiogalactopyranoside (IPTG) induction, but the majority of the PySNAT protein was in an insoluble form (lane 3 in FL of Fig. 2A). In contrast, truncated PySNAT (∆22PySNAT) expression with an N-terminal His-tag enhanced soluble PySNAT expression (lane 3 in ∆22 of Fig. 2A). For the C-terminal His-tagged system, a truncated form of PySNAT showed much higher soluble expression than the full-length form (Fig. 2B). To verify that the purified recombinant *PySNAT* proteins exhibit SNAT enzyme activity, we measured SNAT enzyme activity at 30 °C by measuring *N*-acetylserotonin (NAS) in the presence of serotonin, acetyl-CoA, and various forms of PySNAT proteins. As shown in Fig. 3, all forms of purified recombinant PySNAT proteins contained SNAT catalytic activities, of which the full-length PySNAT with a C-terminal His-tag showed peak SNAT enzyme activity. This suggested that the chloroplast-encoded *PySNAT* gene was a *SNAT* homologue, as found in other plants.

### Enzyme kinetics of the purified recombinant PySNAT protein

**Fig. 4.**
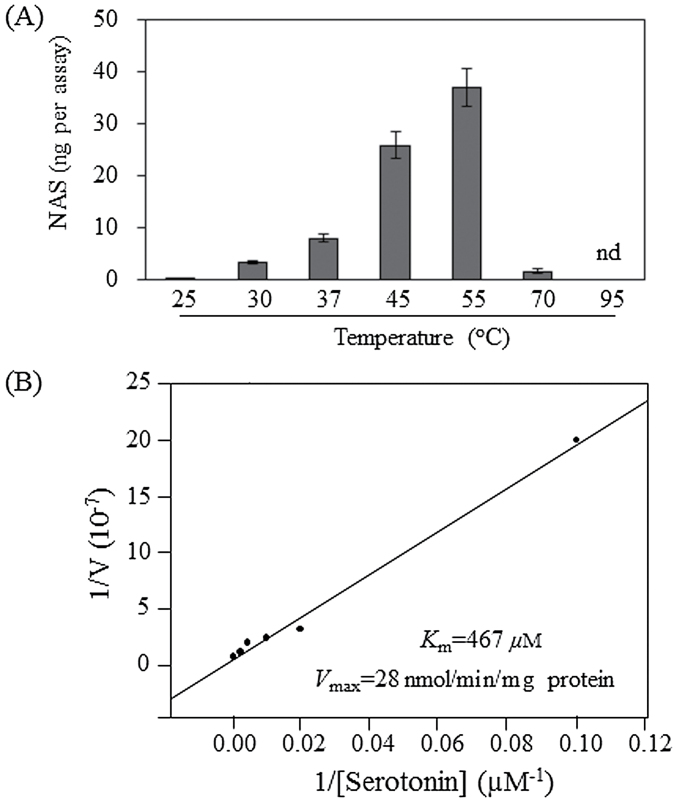
Characterization of the C-terminal His-tagged full-length purified recombinant PySNAT. (A) SNAT enzyme activity as a function of temperature. (B) Determination of *K*_m_ and *V*_max_ of PySNAT for serotonin. PySNAT (1 µg) was incubated at different serotonin concentrations for 30min at 55 °C. The *K*_m_ and *V*_max_ values were determined using a Lineweaver–Burk plot.

We first measured the thermostability of the PySNAT enzyme since plant SNAT proteins (including the cyanobacterial SNAT) show thermophilic characteristics (Byeon *et al.*, 2013, 2014). Purified full-length PySNAT with a C-terminal His-tag was incubated at varying temperatures for the *in vitro* SNAT assay. As shown in Fig. 4A, SNAT enzyme activity increased with temperature. For example, NAS was produced at 0.3ng per assay at 25 °C, whereas 35ng was produced at 55 °C (116-fold increase compared with production at 25 °C). The temperature-dependent rapid increase in SNAT activity is common in plant SNAT enzyme from rice. SNAT enzyme activity peaked at 55 °C, but a rapid decrease in SNAT enzyme activity was observed at 70 °C and no activity occurred at 95 °C. In contrast, the SNAT proteins from rice and cyanobacterum had high activity, even at 70 °C (Byeon *et al.*, 2013, 2014). Thus, we further investigated the enzyme kinetics at the optimal temperature of 55 °C based on Lineweaver–Burk plots. The *K*_m_ and *V*_max_ values were 467 µM and 28 nmol min^–1^ mg^–1^ of protein, respectively, which were 1.8-fold lower and 18-fold higher, respectively, than those for cyanobacterial SNAT. Thus, the catalytic efficiency (*V*_max_/*K*_m_) of PySNAT was 31-fold higher than that of the cyanobacterial SNAT.

### Subcellular localization of PySNAT

**Fig. 5.**
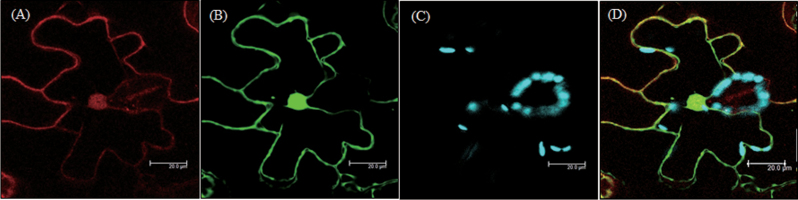
Subcellular localization of PySNAT. (A) Red fluorescence of PySNAT-mCherry. (B) Green fluorescence of cytoplasmic GFP. (C) Cyan fluorescence of chlorophyll. (D) The fluorescence images were merged. *Agrobacterium*-infiltrated tobacco (*Nicotiana benthamiana*) leaves with XVE-inducible *PySNAT*-mCherry were grown for 2 d in a growth room before XVE-induction and visualization by confocal microscopy. Bars, 20 μm. The GenBank accession number of *PySNAT* is NC_007932.

Based on the potential origin of *PySNAT* from cyanobacteria, PySNAT may target to chloroplasts in higher plants because some proteins (even in the absence of chloroplast transit peptides) can localize to chloroplasts (Ha *et al.*, 2003; Jung *et al.*, 2004). To determine whether PySNAT can target to chloroplasts in tobacco, we constructed a binary vector, pER8-PySNAT-mCherry, under the control of the oestrogen-inducible XVE promoter. *Agrobacterium* cells harbouring the binary vector pER8-PySNAT-mCherry were infiltrated into tobacco leaves to examine subcellular localization using confocal microscopy. As shown in Fig. 5, PySANT showed a strong red fluorescence in tobacco cytoplasm (Fig. 5A) that co-localized with the green fluorescence of GFP, a cytoplasmic marker protein (Fig. 5B). The red fluorescence of PySNAT merged with the green fluorescence of cytoplasmic GFP (Fig. 5D), which indicated that the chloroplast-encoded PySNAT is devoid of chloroplast transit peptides and lacks an intrinsic ability to translocate into chloroplasts of higher plants. Although the primary structure of PySNAT (including pI) differed from that of cyanobacteria SNAT, PySNAT itself cannot translocate into chloroplasts in higher plants and requires the acquisition of an N-terminal chloroplast transit peptide during evolution.

### Melatonin levels during the response to high-temperature stress

**Fig. 6.**
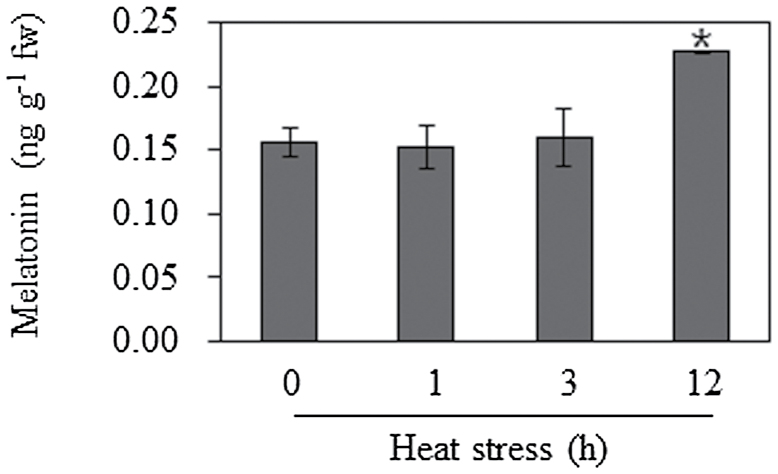
Melatonin levels in response to heat stress in laver (*Pyropia yezoensis*). Leafy gametophytes of *Pyropia yezoensis* were challenged at a high temperature of 25 °C. Laver was collected at each indicated time point. Melatonin levels were quantified by HPLC. Data represent the means±standard deviation of three replicates. An asterisk indicates a significant difference from the wild type (*P*<0.05).

During its natural life cycle, laver is exposed to various adverse environmental stresses such as drought and temperature changes (Ruangchuay and Notoya, 2003; Park *et al.*, 2012*a*). Thus, we exposed laver culture to high temperature (25 °C) *in vitro* to characterize the melatonin response. The melatonin level in control laver was around 0.16ng g^–1^ of fresh weight (fw), and its levels were consistent within 3h after heat stress (Fig. 6). However, melatonin increased to 0.23ng g^–1^ fw at 12h after heat stress, suggestive of an induction of melatonin biosynthesis.

### Acquisition of chloroplast transit peptides during the evolution of plant SNAT proteins

**Fig. 7.**
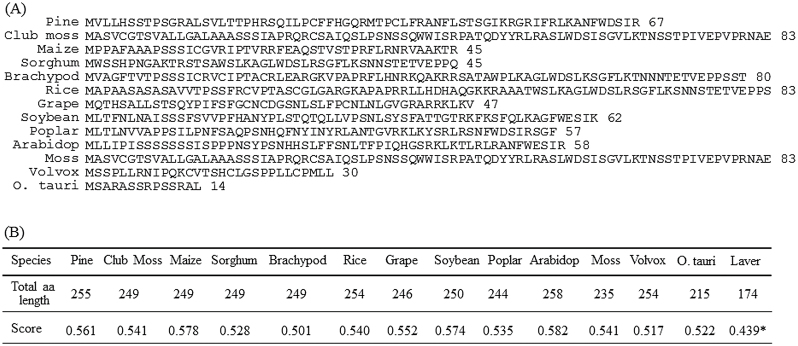
(A) Predicted chloroplast transit peptides of SNAT homologues. (B) Total amino acid lengths of SNAT homologues and ChloroP values. Chloroplast transit peptides were predicted using ChloroP analysis. The sequences included are as follows (GenBank accession numbers): club moss (XP_002983152), maize (NP_001143827), sorghum (XP_002439969), brachypodium (XP_003568235), rice (NP_001055858), grape (XP_002266361), soybean (XP_003534628), poplar (XP_002323094), *Arabidopsis (*NP_001077641), moss (XP_001782491), *Volvox* (XP_002956032), and *O. tauri* (Ot021358). Brachipod, *Brachypodium distachyon*; Arabidop, *Arabidopsis thaliana*; *O. tauri*, *Ostreococcus tauri*. The higher the score, the more confidence that the sequence contains an N-terminal chloroplast transit peptide (Emanuelsson *et al.*, 1999). *This value indicates an absence of the chloroplast transit peptides.

All *SNAT* genes that have been examined in plant lineages possess an N-terminal chloroplast transit peptide according to ChloroP analysis (Emanuelsson *et al.*, 1999). The lengths of chloroplast transit peptides vary among species and range from 14 to 83 aa (Fig. 7). The first acquisition of chloroplast transit peptides occurred in the unicellular green alga *Ostreococcus tauri*, which is thought to possess a 14 aa chloroplast transit peptide. The length of chloroplast transit peptides then increased to 30 aa in the multicellular green alga *Volvox carteri*, although *O. tauri* and *Volvox* belong to Prasinophyceae and Chlorophyceae, respectively, and are not closely related to the ancestors of the embryophytes. However, a moss (*Physcomitrella patens*) positioned between green algae and vascular plants during plant evolution contained an 83 aa chloroplast transit peptide. Other land plants such as maize and rice have various lengths of chloroplast transit peptides ranging from 45 to 83 aa. Thus, the chloroplast transit peptides were probably acquired 1500 million years ago during the evolution of unicellular green algae (Yoon *et al.*, 2004), after which they progressively increased in length until the vascular plants emerged 450 million years ago. The average lengths of the chloroplast transit peptides among plant SNAT proteins was around 58 aa, which is the typical length of average plant chloroplast transit peptides (Zhang and Glaser, 2002). The chloroplast targeting functions of the 14 and 30 aa chloroplast transit peptides in *O. tauri* and *V. carteri*, respectively, requires further analysis.

## Discussion

Melatonin is found in almost all living organisms, including bacteria, animals, and plants, but the level of melatonin in Archaea has not yet been examined (Tan *et al.*, 2012). Due to its ubiquity, melatonin is thought to be a basic component of living organisms (Pandi-Perumal *et al.*, 2006). Melatonin appeared on earth 2.5 billion years ago during the transition from anaerobic to aerobic organisms (Tan *et al.*, 2010). Although melatonin is derived from bacteria in both animals and plants, its bacterial origin differs between animals and plants based on a phylogenetic tree and the evolution of key melatonin biosynthetic genes (Coon and Klein, 2006; Byeon *et al.*, 2013).

SNAT is a key enzyme catalysing the penultimate step in melatonin biosynthesis in both animals and plants. In animals, SNAT is also termed arylalkylamine *N*-acetyltransferase. Given the common ancestor from aerobic bacteria, the *SNAT* gene is likely also to be well conserved in animals and plants. However, a study showed that animal *SNAT* genes have no homology with plant *SNAT* genes (Kang *et al.*, 2013), suggesting that animal and plant *SNAT* genes have evolved independently. Animal *SNAT* homologues were identified in the genomes of Gram-positive bacteria, unicellular green algae, and fungi, suggesting the horizontal transfer of animal *SNAT* from Gram-positive bacteria (Coon and Klein, 2006; Tan *et al.*, 2012). In contrast, plant *SNAT* homologues have been identified in the genomes of cyanobacteria, green algae, moss, gymnosperms, and angiosperms (Byeon *et al.*, 2013; Kang *et al.*, 2013). In addition, the plant *SNAT* homologue was found in the chloroplast genome of the red alga *Pyropia yezoensis*. The chloroplast-encoded red alga *SNAT* had higher SNAT catalytic activity than that of cyanobacterium (Fig. 4B). Collectively, these data suggest that plant *SNAT* was vertically transferred to descendants via endosymbiosis. Considering plant SNAT distribution, the cyanobacteria was thought to have become the chloroplasts in red algae via endosymbiosis, and many plastid-encoded genes including *SNAT* were transferred into the nuclear genome in green algae since gene loss or transfer to the nucleus from plastids is a common phenomenon (Martin and Herrmann, 1998). During the evolution from red algae to green algae and moss, the acquisition of chloroplast transit peptides occurred progressively by extending the length of chloroplast transit peptides (Fig. 7). For example, the unicellular green alga *O. tauri* possessed a 14 aa chloroplast transit peptide, whereas the multicellular green alga *V. carteri* contained a 30 aa chloroplast transit peptide. The moss (*Physcomitrella patens*), phylogenetically close to the ancestors of vascular plants, harboured an 83 aa chloroplast transit peptide. Thus, it is intriguing to examine whether SNAT proteins from *O. tauri* and *V. carteri* target to the chloroplasts, which was observed for rice SNAT (Byeon *et al.*, 2014). Analogous with the chloroplast origin of the plant SNAT gene, plant SNAT clearly localized to chloroplasts (Byeon *et al.*, 2014).

The identification of melatonin in red algae has been reported previously (Hardeland, 1999), in which melatonin levels varied in a red alga (*Pyropia umbilicalis*) grown in the laboratory and in the field (Lorenz and Lüning, 1998). In our report, melatonin levels in *Pyropia yezoensis* were induced in response to heat stress (Fig. 6). This heat-inducible melatonin increase may be associated with an increase in SNAT enzyme activity by high temperature, and not by an increase in *SNAT* mRNA and its translatable polypeptides. The high-temperature-induced melatonin increase was also observed in macroalga and rice (Tal *et al.*, 2011; Byeon and Back, 2014*b*). In addition to high temperature, many abiotic factors are known to induce melatonin expression in plants, including agrochemicals (Vitalini *et al.*, 2011; Park *et al.*, 2013c), plant hormones such as ethylene and abscisic acid (Arnao and Hernández-Ruiz, 2013), high intensity light (Murch *et al.*, 2000; Afreen *et al.*, 2006; Tan *et al.*, 2007*b*; Zhao *et al.*, 2013), high temperature (Tal *et al.*, 2011), senescence (Kang *et al*., 2010), and cold (Murch *et al.*, 2009). Melatonin levels are also increased in specific tissues of plants such as flowers, fruits, seeds, and root tips under normal growth and development (Tan *et al.*, 2007*a*; Hernández-Ruiz and Arnao, 2008; Murch *et al.*, 2009; Okazaki and Ezura, 2009; Ramakrishna *et al.*, 2012; Park *et al.*, 2013*b*; Zhao *et al.*, 2013). Based on a ubiquitous presence and well-conserved biosynthetic gene of melatonin in the plant kingdom, melatonin seems to play a critical role in plant growth and development. However, the role of melatonin in plants requires further study using a knockout mutant plant lacking a key melatonin biosynthetic gene such as *SNAT*. At this time, melatonin-rich transgenic plants through gain-of-function analysis have been generated, which showed similar results to plants challenged with exogenous melatonin treatment (Park and Back, 2012; Park *et al.*, 2013c; Wang *et al.*, 2014). A previous report applied the loss-of-function mutant approach to a melatonin biosynthetic gene, in which the tryptamine 5-hydroxylase gene was deficient or suppressed (Park *et al.*, 2012 *b*, 2013 *a*). However, these mutant lines were melatonin-rich plants rather than melatonin-deficient plants due to the presence of an alternative melatonin biosynthetic pathway in plants.

The *SNAT* genes that have been examined from all land plants and green algae possess chloroplast transit peptides, and their proteins are expected to localize to chloroplasts, which are major sources of reactive oxygen species during photosynthesis. Melatonin or a melatonin precursor such as NAS may play a pivotal role in preventing oxidative damage of chloroplasts by scavenging reactive oxygen species or reactive nitrogen species, resulting in the increase of photosynthesis (Tan *et al*., 2012, 2013; Zhang *et al.*, 2013). However, the exact role and benefit of chloroplast localization of SNAT in plants requires further study.

In summary, we cloned the chloroplast-encoded *PySNAT* gene and measured melatonin levels from the red alga *Pyropia yezoensis*. The purified recombinant PySNAT protein catalysed the conversion of serotonin into NAS at the optimal temperature of 55°C. According to the phylogenetic tree, the red alga *PySNAT* originated from cyanobacteria via endosymbiosis. Thereafter, the chloroplast-encoded SNAT in red alga was transferred into the nuclear genome in green alga through the acquisition of chloroplast transit peptides. Evolution of the plant SNAT gene may follow the direct gene transfer theory from bacteria via an endosymbiotic process.

## Abbreviations:

fw: fresh weight
GFP: green fluorescent protein
HPLC: high-performance liquid chromatography
NAS: *N*-acetylserotonin
SNAT: serotonin *N*-acetyltransferase.
